# Next-generation sequencing: hype and hope for development of personalized radiation therapy?

**DOI:** 10.1186/s13014-015-0481-x

**Published:** 2015-08-28

**Authors:** Ingeborg Tinhofer, Franziska Niehr, Robert Konschak, Sandra Liebs, Matthias Munz, Albrecht Stenzinger, Wilko Weichert, Ulrich Keilholz, Volker Budach

**Affiliations:** Department of Radiooncology and Radiotherapy, Charité University Hospital Berlin, Translational Radiation Oncology Research Laboratory, Charitéplatz 1, 10117 Berlin, Germany; German Cancer Research Center (DKFZ) and German Cancer Consortium (DKTK) partner site, Heidelberg, Germany; Group for Computational Modelling in Medicine, Institute for Theoretical Biology, Humboldt Universität, Berlin, Germany; Institute of Pathology, University Hospital and National Center for Tumor Diseases, Heidelberg, Germany; Institute of Pathology, University Hospital and National Center for Tumor Diseases, Heidelberg, Germany; Charité Comprehensive Cancer Center, Charité University Hospital, Berlin, Germany

## Abstract

The introduction of next-generation sequencing (NGS) in the field of cancer research has boosted worldwide efforts of genome-wide personalized oncology aiming at identifying predictive biomarkers and novel actionable targets. Despite considerable progress in understanding the molecular biology of distinct cancer entities by the use of this revolutionary technology and despite contemporaneous innovations in drug development, translation of NGS findings into improved concepts for cancer treatment remains a challenge. The aim of this article is to describe shortly the NGS platforms for DNA sequencing and in more detail key achievements and unresolved hurdles. A special focus will be given on potential clinical applications of this innovative technique in the field of radiation oncology.

## Introduction

Recent technological advances in DNA sequencing with greater speed and resolution at lower costs has provided new insights in cancer genetics. The next-generation sequencing (NGS) technology is tremendously facilitating the in-depth genome-wide search for genetic alterations which might significantly contribute to aggressive and/or treatment-resistant phenotypes of cancers, thereby establishing the basis for the development of molecularly targeted therapy. High-throughput sequencing of distinct cancer entities in large-scale projects has improved our understanding of the disease-specific mutational patterns [1–4] and the ‘Darwinian’ selection forces involved in subclonal tumor evolution resulting in highly heterogeneous tumors. Initially, NGS has been developed for detection of DNA-based alterations. However, it can also assess other molecular aberrations, including those in the epigenome [5, 6], transcriptome [7, 8] or RNAome [9]. In this review we will only briefly discuss the technical principle of NGS for DNA sequence analysis. For more detailed information we would like to refer the reader to the excellent reviews of Metzker et al. [10], Meyerson et al. [2] and Wong et al. [11]. We will instead focus on key achievements in cancer genetics and potential clinical applications of this innovative technique in the field of radiation oncology.

## The advantages of NGS

Next-generation sequencing has rapidly been evolving within the last decade [10]. This high-throughput method offers several advantages over classical capillary electrophoresis-based ‘Sanger’ sequencing including increased speed and resolution at dramatically lower costs compared to the older sequencing technologies. To illustrate the remarkable progress achieved by NGS, the Human Genome Project which used first-generation ‘Sanger’ sequencing technology to sequence the human genome took over 10 years and nearly 3 billion USD to achieve its goal [12–14]. By next-generation sequencing an individual human genome can now be sequenced in less than 2 weeks for approximately 5000 USD [15].

In theory, the whole genome does not need to be sequenced to identify genetic alterations in most human cancer-associated genes. More than 85 % of pathogenic mutations are found within the protein-coding regions of the genome [16], which collectively are referred to as the “human exome”. This already dramatically reduces the regions that need to be sequenced for personalized oncology, thereby decreasing costs and time for whole exome sequencing of one sample to approximately 1,500 USD and 48 h (the exact prices mainly depend on the NGS platform, the required sequencing depth and are exclusive of the costs for bioinformatics). Furthermore and probably even more relevant for integration into clinical trials [17] or routine diagnostic applications [18], focusing on a selected panel of genes with established impact in cancer progression and/or a proven role in treatment resistance is possible which offers the opportunity for detection of rare genetic variants at very high sensitivity [2, 17] in all types of samples including archival formalin-fixed, paraffin-embedded (FFPE) tissue [18, 19] and plasma cell-free circulating tumor DNA [20].

## The technical principle behind NGS

DNA sequencing was initially developed in 1975 by Sanger and Coulson [21] and these techniques are still used widely today. ‘Sanger’ sequencing is based on the use of oligonucleotide primers specifically binding to either side of the target DNA region which is then amplified in a polymerase chain reaction (PCR). The use of chain-terminating nucleotides in the DNA synthesis process allows the generation of different copies of the original DNA template at all possible lengths, which are separated by capillary electrophoresis. By using specifically labelled chain-terminating nucleotides (A, C, T or G) the original DNA sequence can be assembled.

NGS is based on the principle of sequencing in a massively parallel fashion. This means that up to millions of DNA fragments can be sequenced at the same time. Initially, DNA is fragmented into short segments called a shotgun library. Adaptors are ligated to the ends of each fragment. These adaptors are themselves short sequences of DNA which have primer binding sites for subsequent amplification. The shotgun library can subsequently be enriched for the sequences of interest, using different approaches [22, 23]. As one example, probes which correspond to the target regions, e.g. the human exome, and which are immobilized on beads or a solid plate can be used in order to physically separate the target DNA fragments from the remaining DNA. Alternatively, custom arrays can be designed to enrich for specific groups of genes of interest (cancer gene panels). Following enrichment, the fragment library can be sequenced on next-generation sequencing platforms from several manufacturers (for a comprehensive review of the differing platform techniques see Metzker et al. [10]. Recording of the captured sequences occurs at live mode in a massively parallel fashion when the fluorescent signals from dye-labelled nucleotides in the nascent DNA strands on each bead, channel or cluster are detected during DNA synthesis.

## The challenge of big data analysis from NGS

Whilst large amounts of sequencing data can be generated relatively quickly, data analysis can be time-consuming and difficult. The first problem is the large size of NGS raw data files, especially for results from WES or WGS. For example, non-compressed FASTQ files from human WGS with a mean coverage of 30x requires up to 200 gigabytes, making data transfer and storage of even small WGS projects a real challenge. These estimates do not include the disk space required for any downstream analysis. Development of streamlined, highly automated pipelines for pre-processing of raw data, alignment or de novo assembly of reads, quality control, copy number variation (CNV) and/or SNP calling is essential and high-capacity server solutions are mandatory. The key first step of data processing is the alignment of the sequence reads to a reference genome. Three characteristics of NGS data complicate this task. First, read lengths are relatively short (in average 26–330 bp) [10] compared to capillary-based ‘Sanger’ sequencing, which decreases the likelihood that a read can be mapped to one unique location. Second, reads from NGS platforms contain higher rates of sequencing errors, especially in regions of homopolymer repeats [10]. Subsequent validation of novel variants by ‘Sanger’ sequencing to exclude technical sequencing errors is therefore highly recommended. This technical limitation of NGS is also underlined by the results from a recent study which revealed a higher rate of false-positive single nucleotide variations detected by WES compared to WGS and a considerable fraction of insertions and deletions detected by both WES and WGS which could not be confirmed by subsequent Sanger sequencing [24].

By all means, in each individual case most of the identified variants will represent single nucleotide polymorphisms (SNPs) of no pathogenic relevance [25]. These can be removed either by filtering against sequencing results from ‘control’ DNA of the same patient’s normal tissue or, if such control is not available, against data sets from public databases such as the NCBI dbSNP and the ‘1000-genomes’ project [25]. The remaining variants can be filtered against public collections of genetic alterations in cancer, such as the Catalogue Of Somatic Mutations In Cancer (COSMIC) database (http://cancer.sanger.ac.uk) which as of August 2014 contained over 2 million coding mutations, more than 70,000 gene fusions or genome rearrangements and almost 700,000 abnormal copy number variants [26]. By such an approach, genetic variants with known/potential oncogenic function can be identified.

An additional approach to separate biologically relevant from irrelevant variants often utilizes new software tools (SIFT [27, 28], PolyPhen-2 [29], mutation-assessor [30]) which are now widely available and help to determine which mutations may have a functional impact on the encoded protein, which are likely to be pathogenic, or which are rather neutral variants without biological effect. These methods are generally based on the assumption that important amino acids will be conserved in the protein family, and that changes at well-conserved positions are likely to be deleterious [27]. For example, given a protein sequence SIFT chooses related proteins and obtains an alignment of these proteins with the query. Based on the amino acids appearing at each position in the alignment, SIFT calculates the probability that an amino acid at a position is tolerated or deleterious [27].

MutSig is another algorithm which has been developed at the Broad Institute of Harvard and MIT in 2007 [31]. MutSig is currently broadly used to identify driver mutations among large numbers of passenger mutations. In contrast to the above mentioned methods, MutSig takes into account that background mutation processes occurred during formation of tumors and it considers the mutations of each gene to identify genes that were mutated more often than expected by chance [4]. Besides looking for abundance above background, MutSig looks for positive selection in genes, i.e. increased numbers of non-synonymous vs. silent mutations or mutation clusters at hotspots. Its advanced version (MutSigv2.0) takes also into account the functional impact of mutations (as estimated by the above mentioned tools SIFT, PolypPhen-2, Mutation Assessor, etc). In addition, incorporation of the covariates DNA replication time, chromatin state (open/closed), and general level of transcription activity into the background model has been shown to substantially reduce the number of false-positive findings [4].

These *in-silico* methods certainly assist in the filtering process, however their results still need to be cautiously interpreted in conjunction with the involved gene and certainly have their limitations. Methods like MutSig identifying driver gene mutations based on background mutation rates rely on a correct estimation of this background rate in a given tumor type and at a defined genomic region in order to keep the number of false positives to a minimum [4]. Other algorithms underestimate functional changes in poorly conserved positions [32]. As a result, frequency-based methods with loose background mutation rates will detect driver candidates with a probably high rate of false positives. On the other hand, methods implementing stricter models will identify more specific candidate lists but might miss some true cancer driver genes. Combination of complementary methods might overcome these limitations [3] and will certainly increase the knowledge gain from NGS studies. Last but not least, functional studies in preclinical models for elucidation of the mode of interaction of genetic variants with biological processes in tumor cells are indispensable for validation of NGS findings and are certainly mandatory before NGS technologies should move into clinical applications [33]. Translation into clinical practice can certainly only be achieved by multidisciplinary research approaches in order to extract meaningful diagnostic interpretation from large NGS datasets.

## Novel approaches for personalization of radiotherapy

Over the last two decades, technological advances in treatment planning and delivery have improved the quality of radiotherapy in terms of precise dose application to the target volume together with minimal dose to normal tissue. Despite these achievements, a fundamental question that remains unresolved is whether based on the molecular profile of their tumors it is possible to prospectively identify patients who are more likely to benefit from radiotherapy. Personalized radiotherapy could be achieved by establishing biomarkers which can classify radiosensitive/-resistant tumors and/or tumor-surrounding normal tissue before initiation of treatment. To achieve such goal, previous studies have mostly evaluated single biomarkers or functional assays of DNA damage repair as predictor of intrinsic cellular radiosensitivity. Among others, assessment of the cell survival fraction [34] or the number of residual DNA double strand breaks after *ex vivo* irradiation of tumor cells [35] or normal tissue [36, 37] as well as *in vivo* determination of the extent of tumor hypoxia [38] have been evaluated extensively. Although promising according to preliminary clinical data, none of them have become routine yet which might be due to low robustness of some of these *in-vivo* assays [36].

The generation of high-throughput data sets in the omics era has provided a novel and complementary opportunity in biomarker discovery. Using high-throughput transcriptome analysis, it has been previously shown that prediction of cellular radiosensitivity of tumor cell lines by expression analysis of a defined set of genes clearly outperformed assays of single gene analysis [39]. The value of this molecular signature as predictive biomarker for radiosensitivity was already confirmed in a large clinical cohort [40] speaking for its clinical potential. Another interesting approach is the use of hypoxia gene expression signatures for selecting patients who likely benefit from the inclusion of hypoxia-modifying drugs in regimens of radio- [41] or radiochemotherapy [42].

Beside the influence of gene expression levels, individual differences in cellular radiosensitivity are thought to be at least partly determined by germ-line genetic variants. Rare variants which are likely to be functional can only be detected by high-throughput DNA sequencing, made now affordable by the NGS technology. Up to date, only few studies used NGS for assessment of the exact role of SNPs for treatment outcome after radiotherapy. Recently, the role of germ-line SNPs and rare variants in *MRE11A* as predictive biomarkers of both tumor response and toxicity following definitive radiotherapy of muscle-invasive bladder cancer was analyzed by this technology [43]. Carriers of at least one of six rare *MRE11A* variants had a significantly higher risk of local failure in the radiotherapy arm, whereas no such association was seen in the surgically treated patient cohort [43]. It will certainly be interesting to expand such type of analysis to a broader spectrum of cancer types.

For elucidating the role of somatic mutations in radioresistance NGS has first been applied in bacteria [44]. In a model of cellular adaption to irradiation, extremely radioresistant E.coli strains were generated from the respective founder cells by repetitive cycles of increasing irradiation doses. Whole genome sequencing revealed a large number of genomic alterations in the radioresistant descendants of which only few were recurrent mutations, suggesting that multiple mechanisms can contribute to radiation resistance and distinct evolutionary pathways leading to this phenotype. Intriguingly, despite this heterogeneity, clear genetic patterns also emerged. Not unexpectedly, mutations clustered more frequently in genes of DNA double strand break repair.

In two recent NGS studies in locally advanced squamous cell carcinoma of the head and neck (HNSCC) our group has evaluated the role of somatic mutations in a set of cancer-related genes for the efficacy of definitive [45] and adjuvant chemoradiation [46]. Our studies could confirm previous reports of poor efficacy of radiotherapy in HNSCC tumors harboring disruptive *TP53* mutations [47, 48]. For the first time, we demonstrated a possible role of mutations in *NOTCH1* and key driver genes (*PIK3CA*, *KRAS*, *NRAS* and *HRAS*) as predictive biomarkers of outcome after chemoradiation. Moreover, our studies also confirmed that archival formalin-fixed paraffin-embedded (FFPE) specimens are indeed suitable for targeted NGS although in series older than 8–10 years a considerable portion of samples (up to 30 %) might fail due to the high extent of DNA fragmentation (IT, ms in preparation, July 2015).

NGS is also increasingly being used for the dissection of the mechanisms involved in treatment-induced clonal selection in the course of acquired treatment resistance. To our knowledge, only one study so far has addressed this question in a model of radioresistance [49]. In this study, DNA-targeted sequencing was performed on pre- and post-treatment tumor tissues from rectal cancer patients who failed to respond to neoadjuvant chemoradiation. Mutant variants previously associated with radioresistance including *TP53* were detected in post-treatment residual tumor tissue from non-responders. In line with an important role of *TP53* mutation in radioresistance, an increase in allele frequency of aberrant *TP53* variants as well as an increase in mutant p53 expression levels was observed in all cases in which the tumor harbored a hotspot missense mutation in the DNA-binding domain of p53. These data strongly suggest that chemoradiation exerts a selection pressure that leads to the increase in the relative portion of tumor cells expressing mutant p53 protein [49]. Strategies of downregulating mutant p53 [50] or refolding it into its wild-type confirmation [51] might prove effective in sensitizing tumor cells to chemoradiation in this scenario.

Another interesting approach with potential impact in radiooncology which makes use of NGS represents a novel method named XR-seq. This technique can be applied for genome-wide mapping of DNA excision repair [52]. The underlying principle is that human nucleotide excision repair generates two incisions surrounding the site of damage, creating fragments of approximately 30 nucleotides. In XR-seq, these fragments are enriched by immunoprecipitation of specific repair proteins which are tightly bound to the excised DNA fragments. By subjecting this fragment library to NGS maps of global and transcription-coupled DNA repair can be generated. This novel method will allow uncovering repair characteristics and sequence preferences of treatment-induced DNA damage and as such might facilitate studies of the effects of mutational patterns on transcriptional activity on DNA repair in human tumor cells. This method should also prove useful in determining the effects of drugs like histone-modifying therapeutics or poly ADP ribose polymerase (PARP) inhibitors on nucleotide excision repair, and how they eventually interfere with radio- or chemosensitivity of tumor cells.

The immunomodulatory effects of radiation have been widely documented (for review see Burnette & Weichselbaum [53]) and immunogenic cell death was identified as key component not only of targeted therapies but also conventional treatment modalities including radiation [54]. It could thus be speculated that radiation of tumors with large numbers of genetic alterations, with a portion of them serving as putative neo-antigens, is more likely to induce anti-tumor immunity compared to radiation of tumors with low number of alterations. In support of this assumption, the total number of immunogenic mutations *per se* (identified by WES) was positively correlated with overall survival of cancer patients treated with standard regimens [55]. Combining radiation and immune checkpoint blockade which already demonstrated synergistic anti-tumor responses in animal models [56] are promising strategies which are based on the above-mentioned principles. Integration of NGS-based mutational profiling in upcoming clinical trials of such combinatory treatment are anticipated and will determine the predictive value of the mutational load and/or the number of immunogenic mutations in this setting.

## Intertumoral and intratumoral genomic heterogeneity: a real challenge for personalized medicine

**Fig. 1:**
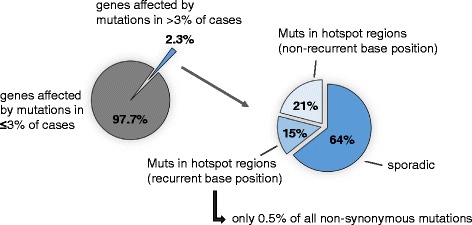
Genetic heterogeneity of squamous cell carcinomas of the head and neck region (HNSCC). The relative distribution of genes affected by mutations is shown according to their mean prevalence within the three analyzed study cohorts (≤3 % vs. >3 % of cases) and their frequency of occurrence at hotspot regions and/or recurrent base positions. The results shown here are based upon somatic mutation data generated by the TCGA Research Network [60], Stransky et al. [59] and Agrawal et al. [58]

As stated above, the technological advances coming along with NGS have permitted rapid analysis of individual cancer genomes at high resolution on single-nucleotide level. By this technical advancement, an astonishing heterogeneity between individual tumors has been revealed, with only a limited number of somatic alterations shared between tumors of the same histopathologic subtype. This large genetic heterogeneity can be illustrated in the model of HNSCC. Cases in this disease entity with a history of heavy smoking and alcohol consumption belong to the group of highly genetically instable tumors [57], most likely resulting from the extensive DNA damage that has been caused by tobacco carcinogen exposure for years. As of December 2014, preprocessed and preanalysed mutational data from 3 independent whole exome NGS studies in HNSCC [58–60] in total reporting on 412 HNSCC cases were available at cbioportal (http://www.cbioportal.org). We used these data which have been filtered using tissue-matched control sequences to exclude germ-line variants for a more detailed assessment of the extent of genetic heterogeneity in HNSCC. Overall, somatic non-synonymous mutations were detected in 15,293 genes. However, only 357 (2.3 %) of these genes were altered by mutation in >3 % of the tumors. In 127 (36 %) of the more frequently affected genes the mutation occurred within hotspot regions but for only 75 genes (15 %) the same base position was involved in more than one tumors. This means that recurrent mutations at hotspot regions were detected in only 0.5 % of all genes altered by mutations (Fig. 1). Alternatively, when the non-synonymous mutations were filtered using the MutSigv2.0 algorithm according to the background mutational rate per gene rather than their prevalence in HNSCC, only 51 genes (0.3 % of all affected genes) were identified as significantly mutated genes.

A second example for tumors of very high genetic heterogeneity is cutaneous melanoma [4]. In a landmark WES study on paired tumor and normal genomic DNA from 135 patients with melanoma an overall number of 86,813 coding mutations were detected at a 2:1 ratio of non-synonymous to synonymous events, suggestive for a high passenger mutation load [61]. Filtering against the basal mutation rates using MutSig [31] produced a list of 544 significantly mutated genes. By refining the algorithm to select for non-synonymous mutations of predicted functional consequence the authors reduced the list of candidate drivers to eleven genes harboring significant functional mutation burden. Interestingly, these genes included six well-known cancer genes (BRAF, NRAS, PTEN, TP53, CDKN2A, MAP2K1) and five new candidates (PPP6C, RAC1, SNX31, TACC1, and STK19) [61].

The huge genetic heterogeneity in these types of cancer underlines the need for advanced bioinformatics models for data analysis. It also impressively illustrates the need of identifying key oncogenic driver pathways rather than individual genes as targets of precision medicine. This assumption is also supported by the observation that many low-frequency mutations in breast and colorectal tumors, each of them having small effects on cell survival [62]. It is thus rather unlikely that genome sequencing will uncover a single target as the “Achilles heel” of a tumor.

Exacerbating the complexity of the genetic landscape of tumors, intratumoral heterogeneity in terms of spatial and temporal differences in the mutational patterns of key driver genes has recently been demonstrated for renal [63, 64], lung [65], colorectal [66, 67] and breast cancer [68]. Beyond etiologic, microenvironmental and tumor-specific factors which all might contribute to such genetic heterogeneity, therapy may act as further exogenous source of genome instability. Consistent with this, in a recent study using the genetic model system Caenorhabditis elegans cisplatin treatment has been found to lead to a striking increase in base substitutions as well as an elevated rate of larger structural alterations [69]. Importantly, among the mutations found to be induced by cisplatin in the human model some variants have been linked to tumor progression and drug resistance like activating *HRAS* mutations at codons 12 and 13 [70, 71]. Temozolomide which is broadly used as radiosensitizer in brain tumors and sarcomas has been found to leave an imprint in the cancer genome in the form of an elevated rate of C > T transitions [57]. Concerning potential mutagenicity of radiotherapy, *TP53* [72] as well as c-*MYC* among others were identified as radiosensitive gene loci [73].

In the light of accumulating evidence for high inter- and intratumoral genomic heterogeneity the identification of the relevant driver mutation(s) among passengers in an individual cancer biopsy at a defined stage of disease represents a significant hurdle in the development of NGS-based molecular diagnostics and personalized treatment. One approach to overcome such hurdle might represent deep sequencing of cell-free circulating tumor DNA derived from blood plasma for personalized cancer genomic profiling [20, 74–78], assuming that genetic variants which are present in tumors only at subclonal level (and which are probably not captured by the diagnostic biopsy) are finally and inevitably released by dying tumor cells to this common reservoir.

## Future perspectives

Exciting new data from a continuously growing number of NGS cancer studies nourish the hope that this technology will also significantly contribute to increasing our understanding of the molecular mechanisms of radioresistance. However, many more studies will certainly be needed to determine the functional consequences of individual mutations or distinct mutational patterns for cellular radiosensitivity and the individual tumor’s response to radiotherapy. Proteomics is expected to provide additional important information that will guide candidate drug selection and recent advances in proteomic techniques [79, 80] have opened new avenues for optimized cancer treatment. The application of these techniques will not only allow the monitoring of protein-protein interactions, posttranslational modification and drug-target engagement directly in cells or tissues but will also represent a valuable tool for identifying off-target drug effects [80]. The latter feature will certainly also foster attempts to develop less toxic protocols of radiotherapy combined with molecularly targeted radiosensitizing agents.

The future of personalized radiation therapy will most likely not only include DNA-based NGS. It will also apply other high-throughput technologies such as RNA sequencing that in parallel provides quantitative gene expression as well as mutational status. Overall, it can be reasoned that integration of mutational patterns from NGS analysis and other omics data together with functional measures of cellular radiosensitivity in systems biology models will strongly improve the power of outcome prediction and optimize current treatment selection algorithms for individual patients.

## References

[1] Ciriello G, Miller ML, Aksoy BA, Senbabaoglu Y, Schultz N, Sander C (2013). Emerging landscape of oncogenic signatures across human cancers. Nat Genet.

[2] Meyerson M, Gabriel S, Getz G (2010). Advances in understanding cancer genomes through second-generation sequencing. Nat Rev Genet.

[3] Tamborero D, Gonzalez-Perez A, Perez-Llamas C, Deu-Pons J, Kandoth C, Reimand J (2013). Comprehensive identification of mutational cancer driver genes across 12 tumor types. Sci Rep..

[4] Lawrence MS, Stojanov P, Polak P, Kryukov GV, Cibulskis K, Sivachenko A (2013). Mutational heterogeneity in cancer and the search for new cancer-associated genes. Nature.

[5] Ku CS, Naidoo N, Wu M, Soong R (2011). Studying the epigenome using next generation sequencing. J Med Genet.

[6] Sarda S, Hannenhalli S (2014). Next-generation sequencing and epigenomics research: a hammer in search of nails. Genomics Inform.

[7] Morozova O, Hirst M, Marra MA (2009). Applications of new sequencing technologies for transcriptome analysis. Annu Rev Genomics Hum Genet..

[8] Wang Z, Gerstein M, Snyder M (2009). RNA-Seq: a revolutionary tool for transcriptomics. Nat Rev Genet.

[9] Derks KW, Misovic B, van den Hout MC, Kockx CE, Gomez CP, Brouwer RW (2015). Deciphering the RNA landscape by RNAome sequencing. RNA Biol.

[10] Metzker ML (2010). Sequencing technologies - the next generation. Nat Rev Genet.

[11] Wong KM, Hudson TJ, McPherson JD (2011). Unraveling the genetics of cancer: genome sequencing and beyond. Annu Rev Genomics Hum Genet..

[12] Lander ES, Linton LM, Birren B, Nusbaum C, Zody MC, Baldwin J (2001). Initial sequencing and analysis of the human genome. Nature.

[13] McPherson JD, Marra M, Hillier L, Waterston RH, Chinwalla A, Wallis J (2001). A physical map of the human genome. Nature.

[14] Sachidanandam R, Weissman D, Schmidt SC, Kakol JM, Stein LD, Marth G (2001). A map of human genome sequence variation containing 1.42 million single nucleotide polymorphisms. Nature.

[15] Wetterstrand KA. DNA Sequencing Costs: Data from the NHGRI Genome Sequencing Program (GSP) Available at: www.genome.gov/sequencingcosts. Accessed [2015].

[16] Ng SB, Turner EH, Robertson PD, Flygare SD, Bigham AW, Lee C (2009). Targeted capture and massively parallel sequencing of 12 human exomes. Nature.

[17] Simon R, Roychowdhury S (2013). Implementing personalized cancer genomics in clinical trials. Nat Rev Drug Discov.

[18] Kriegsmann M, Endris V, Wolf T, Pfarr N, Stenzinger A, Loibl S (2014). Mutational profiles in triple-negative breast cancer defined by ultradeep multigene sequencing show high rates of PI3K pathway alterations and clinically relevant entity subgroup specific differences. Oncotarget.

[19] Hedegaard J, Thorsen K, Lund MK, Hein AM, Hamilton-Dutoit SJ, Vang S (2014). Next-generation sequencing of RNA and DNA isolated from paired fresh-frozen and formalin-fixed paraffin-embedded samples of human cancer and normal tissue. PLoS One.

[20] Lebofsky R, Decraene C, Bernard V, Kamal M, Blin A, Leroy Q (2015). Circulating tumor DNA as a non-invasive substitute to metastasis biopsy for tumor genotyping and personalized medicine in a prospective trial across all tumor types. Mol Oncol.

[21] Sanger F, Coulson AR (1975). A rapid method for determining sequences in DNA by primed synthesis with DNA polymerase. J Mol Biol.

[22] Johansson H, Isaksson M, Sorqvist EF, Roos F, Stenberg J, Sjoblom T (2011). Targeted resequencing of candidate genes using selector probes. Nucleic Acids Res.

[23] Bodi K, Perera AG, Adams PS, Bintzler D, Dewar K, Grove DS (2013). Comparison of commercially available target enrichment methods for next-generation sequencing. J Biomol Tech.

[24] Belkadi A, Bolze A, Itan Y, Cobat A, Vincent QB, Antipenko A (2015). Whole-genome sequencing is more powerful than whole-exome sequencing for detecting exome variants. Proc Natl Acad Sci U S A.

[25] Genomes Project C, Abecasis GR, Altshuler D, Auton A, Brooks LD, Durbin RM (2010). A map of human genome variation from population-scale sequencing. Nature.

[26] Forbes SA, Beare D, Gunasekaran P, Leung K, Bindal N, Boutselakis H (2015). COSMIC: exploring the world’s knowledge of somatic mutations in human cancer. Nucleic Acids Res.

[27] Ng PC, Henikoff S (2003). SIFT: Predicting amino acid changes that affect protein function. Nucleic Acids Res.

[28] Kumar P, Henikoff S, Ng PC (2009). Predicting the effects of coding non-synonymous variants on protein function using the SIFT algorithm. Nat Protoc.

[29] Adzhubei IA, Schmidt S, Peshkin L, Ramensky VE, Gerasimova A, Bork P (2010). A method and server for predicting damaging missense mutations. Nat Methods.

[30] Reva B, Antipin Y, Sander C (2011). Predicting the functional impact of protein mutations: application to cancer genomics. Nucleic Acids Res.

[31] Getz G, Hofling H, Mesirov JP, Golub TR, Meyerson M, Tibshirani R (2007). Comment on “The consensus coding sequences of human breast and colorectal cancers”. Science.

[32] Reimand J, Wagih O, Bader GD (2013). The mutational landscape of phosphorylation signaling in cancer. Sci Rep..

[33] Schott AF, Perou CM, Hayes DF (2015). Genome Medicine in Cancer: What’s in a Name?. Cancer Res.

[34] Pouliliou SE, Lialiaris TS, Dimitriou T, Giatromanolaki A, Papazoglou D, Pappa A (2015). Survival Fraction at 2 Gy and gammaH2AX Expression Kinetics in Peripheral Blood Lymphocytes From Cancer Patients: Relationship With Acute Radiation-Induced Toxicities. Int J Radiat Oncol Biol Phys.

[35] Menegakis A, Eicheler W, Yaromina A, Thames HD, Krause M, Baumann M (2011). Residual DNA double strand breaks in perfused but not in unperfused areas determine different radiosensitivity of tumours. Radiother Oncol.

[36] van Waarde MA, van Assen AJ, Konings AW, Kampinga HH (1996). Feasibility of measuring radiation-induced DNA double strand breaks and their repair by pulsed field gel electrophoresis in freshly isolated cells from the mouse RIF-1 tumor. Int J Radiat Oncol Biol Phys.

[37] Rube CE, Grudzenski S, Kuhne M, Dong X, Rief N, Lobrich M (2008). DNA double-strand break repair of blood lymphocytes and normal tissues analysed in a preclinical mouse model: implications for radiosensitivity testing. Clin Cancer Res.

[38] Zips D, Eicheler W, Bruchner K, Jackisch T, Geyer P, Petersen C (2001). Impact of the tumour bed effect on microenvironment, radiobiological hypoxia and the outcome of fractionated radiotherapy of human FaDu squamous-cell carcinoma growing in the nude mouse. Int J Radiat Biol.

[39] Eschrich S, Zhang H, Zhao H, Boulware D, Lee JH, Bloom G (2009). Systems biology modeling of the radiation sensitivity network: a biomarker discovery platform. Int J Radiat Oncol Biol Phys.

[40] Eschrich SA, Fulp WJ, Pawitan Y, Foekens JA, Smid M, Martens JW (2012). Validation of a radiosensitivity molecular signature in breast cancer. Clin Cancer Res.

[41] Toustrup K, Sorensen BS, Lassen P, Wiuf C, Alsner J, Overgaard J (2012). Gene expression classifier predicts for hypoxic modification of radiotherapy with nimorazole in squamous cell carcinomas of the head and neck. Radiother Oncol.

[42] Hassan Metwally MA, Ali R, Kuddu M, Shouman T, Strojan P, Iqbal K, et al. IAEA-HypoX. A randomized multicenter study of the hypoxic radiosensitizer nimorazole concomitant with accelerated radiotherapy in head and neck squamous cell carcinoma. Radiother Oncol. 2015. 10.1016/j.radonc.2015.04.005 25913070

[43] Teo MT, Dyrskjot L, Nsengimana J, Buchwald C, Snowden H, Morgan J (2014). Next-generation sequencing identifies germline MRE11A variants as markers of radiotherapy outcomes in muscle-invasive bladder cancer. Ann Oncol.

[44] Harris DR, Pollock SV, Wood EA, Goiffon RJ, Klingele AJ, Cabot EL (2009). Directed evolution of ionizing radiation resistance in Escherichia coli. J Bacteriol.

[45] Tinhofer I, Budach V, Endris V, Stenzinger A, Weichert W (2014). Genomic profiling using targeted ultra-deep next-generation sequencing for prediction of treatment outcome after concurrent chemoradiation: Results from the German ARO-0401 trial. J Clin Oncol.

[46] Tinhofer I, Budach V, Linge A, Lohaus F, Gkika E, Stuschke M (2015). Mutational patterns of HPV+ and HPV- squamous cell carcinomas of the head and neck (SCCHN) and their interference with outcome after adjuvant chemoradiation: A multicenter biomarker study of the German Cancer Consortium Radiation Oncology Group. J Clin Oncol..

[47] Lindenbergh-van der Plas M, Brakenhoff RH, Kuik DJ, Buijze M, Bloemena E, Snijders PJ (2011). Prognostic significance of truncating TP53 mutations in head and neck squamous cell carcinoma. Clin Cancer Res.

[48] Skinner HD, Sandulache VC, Ow TJ, Meyn RE, Yordy JS, Beadle BM (2012). TP53 disruptive mutations lead to head and neck cancer treatment failure through inhibition of radiation-induced senescence. Clin Cancer Res.

[49] Sakai K, Kazama S, Nagai Y, Murono K, Tanaka T, Ishihara S (2014). Chemoradiation provides a physiological selective pressure that increases the expansion of aberrant TP53 tumor variants in residual rectal cancerous regions. Oncotarget.

[50] Rodriguez OC, Choudhury S, Kolukula V, Vietsch EE, Catania J, Preet A (2012). Dietary downregulation of mutant p53 levels via glucose restriction: mechanisms and implications for tumor therapy. Cell Cycle.

[51] Bykov VJ, Issaeva N, Selivanova G, Wiman KG (2002). Mutant p53-dependent growth suppression distinguishes PRIMA-1 from known anticancer drugs: a statistical analysis of information in the National Cancer Institute database. Carcinogenesis.

[52] Hu J, Adar S, Selby CP, Lieb JD, Sancar A (2015). Genome-wide analysis of human global and transcription-coupled excision repair of UV damage at single-nucleotide resolution. Genes Dev.

[53] Burnette B, Weichselbaum RR (2013). Radiation as an immune modulator. Semin Radiat Oncol.

[54] Kroemer G, Galluzzi L, Kepp O, Zitvogel L (2013). Immunogenic cell death in cancer therapy. Annu Rev Immunol..

[55] Brown SD, Warren RL, Gibb EA, Martin SD, Spinelli JJ, Nelson BH (2014). Neo-antigens predicted by tumor genome meta-analysis correlate with increased patient survival. Genome Res.

[56] Binder DC, Fu YX, Weichselbaum RR. Radiotherapy and immune checkpoint blockade: potential interactions and future directions. Trends Mol Med. 2015 10.1016/j.molmed.2015.05.007 26091823

[57] Alexandrov LB, Nik-Zainal S, Wedge DC, Aparicio SA, Behjati S, Biankin AV (2013). Signatures of mutational processes in human cancer. Nature.

[58] Agrawal N, Frederick MJ, Pickering CR, Bettegowda C, Chang K, Li RJ (2011). Exome sequencing of head and neck squamous cell carcinoma reveals inactivating mutations in NOTCH1. Science.

[59] Stransky N, Egloff AM, Tward AD, Kostic AD, Cibulskis K, Sivachenko A (2011). The mutational landscape of head and neck squamous cell carcinoma. Science.

[60] The Cancer Genome Atlas N (2015). Comprehensive genomic characterization of head and neck squamous cell carcinomas. Nature..

[61] Hodis E, Watson IR, Kryukov GV, Arold ST, Imielinski M, Theurillat JP (2012). A landscape of driver mutations in melanoma. Cell.

[62] Wood LD, Parsons DW, Jones S, Lin J, Sjoblom T, Leary RJ (2007). The genomic landscapes of human breast and colorectal cancers. Science.

[63] Gerlinger M, Rowan AJ, Horswell S, Larkin J, Endesfelder D, Gronroos E (2012). Intratumor heterogeneity and branched evolution revealed by multiregion sequencing. N Engl J Med.

[64] Swanton C (2012). Intratumor heterogeneity: evolution through space and time. Cancer Res.

[65] de Bruin EC, McGranahan N, Mitter R, Salm M, Wedge DC, Yates L (2014). Spatial and temporal diversity in genomic instability processes defines lung cancer evolution. Science.

[66] Baldus SE, Schaefer KL, Engers R, Hartleb D, Stoecklein NH, Gabbert HE (2010). Prevalence and heterogeneity of KRAS, BRAF, and PIK3CA mutations in primary colorectal adenocarcinomas and their corresponding metastases. Clin Cancer Res.

[67] Kreso A, O’Brien CA, van Galen P, Gan OI, Notta F, Brown AM (2013). Variable clonal repopulation dynamics influence chemotherapy response in colorectal cancer. Science.

[68] Navin N, Kendall J, Troge J, Andrews P, Rodgers L, McIndoo J (2011). Tumour evolution inferred by single-cell sequencing. Nature.

[69] Meier B, Cooke SL, Weiss J, Bailly AP, Alexandrov LB, Marshall J (2014). C. elegans whole-genome sequencing reveals mutational signatures related to carcinogens and DNA repair deficiency. Genome Res.

[70] Munoz EF, Diwan BA, Calvert RJ, Weghorst CM, Anderson J, Rice JM (1996). Transplacental mutagenicity of cisplatin: H-ras codon 12 and 13 mutations in skin tumors of SENCAR mice. Carcinogenesis.

[71] Cho HJ, Jeong HG, Lee JS, Woo ER, Hyun JW, Chung MH (2002). Oncogenic H-Ras enhances DNA repair through the Ras/phosphatidylinositol 3-kinase/Rac1 pathway in NIH3T3 cells. Evidence for association with reactive oxygen species. J Biol Chem.

[72] Gonin-Laurent N, Gibaud A, Huygue M, Lefevre SH, Le Bras M, Chauveinc L (2006). Specific TP53 mutation pattern in radiation-induced sarcomas. Carcinogenesis.

[73] Wade MA, Sunter NJ, Fordham SE, Long A, Masic D, Russell LJ, et al. c-MYC is a radiosensitive locus in human breast cells. Oncogene. 2014. 10.1038/onc.2014.427 PMC4391966 25531321

[74] Forshew T, Murtaza M, Parkinson C, Gale D, Tsui DW, Kaper F (2012). Noninvasive identification and monitoring of cancer mutations by targeted deep sequencing of plasma DNA. Sci Transl Med.

[75] Dawson SJ, Tsui DW, Murtaza M, Biggs H, Rueda OM, Chin SF (2013). Analysis of circulating tumor DNA to monitor metastatic breast cancer. N Engl J Med.

[76] Bettegowda C, Sausen M, Leary RJ, Kinde I, Wang Y, Agrawal N (2014). Detection of circulating tumor DNA in early- and late-stage human malignancies. Sci Transl Med.

[77] Newman AM, Bratman SV, To J, Wynne JF, Eclov NC, Modlin LA (2014). An ultrasensitive method for quantitating circulating tumor DNA with broad patient coverage. Nat Med.

[78] Frenel JS, Carreira S, Goodall J, Roda Perez D, Perez Lopez R, Tunariu N, et al. Serial Next Generation Sequencing of Circulating Cell Free DNA Evaluating Tumour Clone Response To Molecularly Targeted Drug Administration. Clin Cancer Res. 2015. 10.1158/1078-0432.CCR-15-0584 PMC4580992 26085511

[79] Martinez Molina D, Jafari R, Ignatushchenko M, Seki T, Larsson EA, Dan C (2013). Monitoring drug target engagement in cells and tissues using the cellular thermal shift assay. Science.

[80] Savitski MM, Reinhard FB, Franken H, Werner T, Savitski MF, Eberhard D (2014). Tracking cancer drugs in living cells by thermal profiling of the proteome. Science.

